# Expert consensus on digital guided therapy for endodontic diseases

**DOI:** 10.1038/s41368-023-00261-0

**Published:** 2023-12-06

**Authors:** Xi Wei, Yu Du, Xuedong Zhou, Lin Yue, Qing Yu, Benxiang Hou, Zhi Chen, Jingping Liang, Wenxia Chen, Lihong Qiu, Xiangya Huang, Liuyan Meng, Dingming Huang, Xiaoyan Wang, Yu Tian, Zisheng Tang, Qi Zhang, Leiying Miao, Jin Zhao, Deqin Yang, Jian Yang, Junqi Ling

**Affiliations:** 1 Department of Operative Dentistry and Endodontics, Hospital of Stomatology, Guanghua, School of Stomatology, Sun Yat-Sen University & Guangdong Provincial Key Laboratory of Stomatology, Guangzhou, China; 2 State Key Laboratory of Oral Diseases & National Center for Stomatology & National Clinical Research Center for Oral Diseases & Department of Cariology and Endodontics, West China Hospital of Stomatology, Sichuan University, Chengdu, China; 3 Department of Cariology and Endodontology, Peking University School and Hospital of Stomatology & National Center of Stomatology & National Clinical Research Center for Oral Diseases & National Engineering Laboratory for Digital and Material Technology of Stomatology & Beijing Key Laboratory of Digital Stomatology & Research Center of Engineering and Technology for Computerized Dentistry Ministry of Health & NMPA Key Laboratory for Dental Materials, Beijing, China; 4 Department of Operative Dentistry & Endodontics, School of Stomatology, The Fourth Military Medical University, Xi’an, China; 5 Department of Endodontics, Beijing Stomatological Hospital, School of Stomatology, Capital Medical University, Beijing, China; 6 The State Key Laboratory Breeding Base of Basic Science of Stomatology (Hubei-MOST) & Key Laboratory of Oral Biomedicine Ministry of Education, School and Hospital of Stomatology, Wuhan University, Wuhan, China; 7 Department of Endodontics, Shanghai Ninth People’s Hospital, Shanghai Jiao Tong University School of Medicine; College of Stomatology, Shanghai Jiao Tong University; National Clinical Research Center for Oral Diseases; National Center for Stomatology; Shanghai Key Laboratory of Stomatology, Shanghai, China; 8 College of Stomatology, Hospital of Stomatology, Guangxi Medical University, Nanning, China; 9 Department of Endodontics, School of Stomatology, China Medical University, Shenyang, China; 10 Department of Stomatology, Xinhua Hospital, Shanghai Jiaotong University School of Medicine, Shanghai, China; 11 Department of Endodontics, Stomatological Hospital and Dental School of Tongji University, Shanghai Engineering Research Center of Tooth Restoration and Regeneration, Shanghai, China; 12 Department of Cariology and Endodontics, Nanjing Stomatological Hospital, Medical School of Nanjing University, Nanjing, China; 13 Department of Endodontics, First Affiliated Hospital of Xinjiang Medical University, and College of Stomatology of Xinjiang Medical University, Urumqi, China; 14 Department of Endodontics, Stomatological Hospital of Chongqing Medical University, Chongqing, China; 15 Department of Endodontics, The Affiliated Stomatological Hospital of Nanchang University, Nanchang, China

**Keywords:** Oral diseases, Health care

## Abstract

Digital guided therapy (DGT) has been advocated as a contemporary computer-aided technique for treating endodontic diseases in recent decades. The concept of DGT for endodontic diseases is categorized into static guided endodontics (SGE), necessitating a meticulously designed template, and dynamic guided endodontics (DGE), which utilizes an optical triangulation tracking system. Based on cone-beam computed tomography (CBCT) images superimposed with or without oral scan (OS) data, a virtual template is crafted through software and subsequently translated into a 3-dimensional (3D) printing for SGE, while the system guides the drilling path with a real-time navigation in DGE. DGT was reported to resolve a series of challenging endodontic cases, including teeth with pulp obliteration, teeth with anatomical abnormalities, teeth requiring retreatment, posterior teeth needing endodontic microsurgery, and tooth autotransplantation. Case reports and basic researches all demonstrate that DGT stand as a precise, time-saving, and minimally invasive approach in contrast to conventional freehand method. This expert consensus mainly introduces the case selection, general workflow, evaluation, and impact factor of DGT, which could provide an alternative working strategy in endodontic treatment.

## Introduction

Root canal therapy (RCT) and endodontic microsurgery (EMS) are common treatments for managing endodontic diseases. Through the synergistic utilization of dental operating microscopy (DOM), ultrasonic tips, cone-beam computed tomography (CBCT), and modern filling materials, the pooled success rates of contemporary RCT and EMS were estimated to be 92.6% and 91.3%, respectively.^1,2^ However, searching for root canals in the cases with pulp canal obliteration (PCO) or anatomical abnormalities remains to be an expert-dependent and time-consuming task in clinical practice. Numerous factors including dental trauma, caries, aging, abrasion, pulp capping, and orthodontic treatment may trigger PCO, leading to the deposition of mineralized tissue in the root canal space.^3–6^ More than 25% of these cases may develop into pulp necrosis with radiographic signs of periapical disease and thus need to undergo RCT.^7,8^ However, the exploration of these obliterated canals presents a considerable risk of causing excessive tooth structural loss or perforation.^9^ Similarly, pinpointing the precise location of the root apex during EMS is also troubled where a thick buccal plate and anatomical obstacles like the mental foramen or maxillary sinus are present, potentially compromising the prognosis.^10–12^ Therefore, more precise and minimally invasive strategies need to be introduced to those complicated cases during the operation of RCT or EMS.

## Background And Definition

The inspiration of digital navigation therapy (DGT) for endodontic diseases was developed from guided implantology, and it was first raised as a novel concept as guided endodontics (GE) to gain access to root canals through computer-designed templates to root canals by Krastl et al.^13^ and Zehnder et al.^14^ in 2016. In fact, prior case reports of Dens invaginatus (DI) has already applied guides to indicate the optimal penetration point and drilling direction that allow to access the invagination space or pulp chamber.^15,16^ At the beginning of DGT, a CBCT and an oral scan (OS) were performed and matched through software to facilitate virtual drill planning, then the templates were fabricated by a 3-dimensional (3D) printer. After positioning the templates on the tooth, a specific drill bur was used to gain access to the root canals. The subsequent procedures of root canal instrumentation, irrigation, and obturation were performed routinely by clinicians. The case report and the basic research both indicated GE as a safe and clinically feasible approach to locate root canals.^13,14^ Since then, booming studies have been testified to achieve access cavity preparation by GE in anterior teeth or even posterior teeth.^17–20^ The terminology of GE was also referred as microguided endodontics in some publications.^17,18^ Simultaneously, surgical template was also applied in EMS for guided osteotomy and root apex resection.^21^ Giacomino et al.^22^ used surgical guides and trephine burs to achieve single-step osteotomy, root-end resection, and biopsy during EMS, which was introduced as targeted EMS (TEMS). Subsequent studies claimed that TEMS increased the predictability of EMS, and efficiently minimize the risk of intraoperative complications or postoperative sequelae.^23–25^

In this article, GE, microguided endodontics, and TEMS are all defined as DGT due to their shared foundational principles. However, DGT using guide have inevitable limitations, such as the additional treatment time, the supplementary cost for template fabrication, the absence of real-time visualization, and inability to change. Consequently, dynamic guidance systems (DNS) facilitating dental implantology were introduced since 2019.^26,27^ Afterwards, the definition of DGT for endodontic diseases was further categorized into static GE (SGE) and dynamic GE (DGE). As compared to SGE which requires to use templates, DGE allows clinicians to visualize the position, depth, and angulation of access preparation or osteotomy, which can be adjusted in real time. By operating different DNS, DGE also helps to treat PCO and EMS cases with less iatrogenic errors in a short time.^26,28,29^

To date, more than 150 articles are found at PubMed when searching with the keywords “guided endodontics”, “microguided endodontics”, “targeted endodontic microsurgery”, “static guided endodontics”, “dynamic guided endodontics”, and “dynamic navigation endodontics”. These articles encompass a diverse range of types, including basic research, case reports, case series, retrospective studies, and reviews, but the majority of which are case reports and basic researches. The indications of DGT include RCT, root canal retreatment, EMS, and tooth autotransplantation. Despite two research groups have described the workflow of TEMS and GE,^30,31^ no official guideline has been published yet. The aim of this expert consensus is to summarize the evidence on those techniques to provide an appropriated guidance of DGT for clinical endodontic practice.

## Case selection

### RCT

#### Teeth with obliterated canals

RCT is not necessary in most of the PCO cases because they are asymptomatic. PCO is often noticed incidentally by discoloration of the tooth crown or a radiographic examination. Only when clinical symptoms or radiographic periapical lesions occur, RCT is suggested.^7,8^ However, the calcified tissues block the canal access and thus make RCT difficult for both inexperienced or experienced endodontists.^32^

Based on the reported studies, utilizing DGT strategy in PCO cases should be firstly recommended on anterior teeth with single straight roots and clear signs of apical periodontitis. When most of the canals could be visualized in the apical third of roots from CBCT, designed template may efficiently guide the specific bur to penetrate through the obliterated part of the root canal and obtain access to the apical part.^13,17^

It should be noticed that due to the purpose to get a straight-line access, the drilling in most DGT cases compromised the incisal edges of the anterior teeth.^13,17,33–35^ Although previous SGE study designed multiple drilling guides, including enamel guide and dentin guide to perform access palatally,^36^ it increased the cost of templates and complexity of treatment. By appropriate enamel removal in advance and real-time adjust, DGE has demonstrated the feasibility of attaining a conventional palatal access in our report,^37^ but the drilling process may lack stability without the support of templates.

Whether DGT could be used to locate calcified canals of posterior teeth remains controversial. Although both SGE and DGE have been tried on premolars and molars,^19,28,38,39^ the dentural location, interocclusal distance, and curved canals still provides a big challenge to clinicians.

#### Teeth with anatomical abnormalities

Human dental anomalies include tooth agenesis, hypodontia, delayed tooth formation or eruption, tooth with anatomical abnormalities, and supernumerary teeth.^40^ So far, there are a few case reports about the application of SGE on teeth with anatomical abnormalities.

DI is a relatively common anatomical abnormality with an overall prevalence at 9% in the adult population assessed by CBCT.^41^ A CBCT survey in Chinese population showed that DI has a prevalence of 8.47% and a tooth prevalence of 0.494%.^42^ The morphology of DI varies, which may be normal, conical shape, plug shape, talon cusp shape, and grooves. Usually, the diagnosis of DI relies on the radiographic examination, especially CBCT which could provide 3D images.^43^ The most widely accepted classification of DI was proposed by Oehlers^44^ in 1957: Type I, the invagination not extending beyond the cementoenamel junction. Type II, the invagination that invades the root but remains confined as a blind sac. Type III, an invagination that extends beyond the cementoenamel junction and communicates directly with the periodontal ligament laterally (Type IIIa) or at the apical foramen (Type IIIb).

The treatment plan of DI hinges on its individual anatomy, pulp vitality, periapical state, periodontal state, and source of sinus tract.^45^ Infolding of enamel into dentin forms irregular root canal system, which leads difficulty when performing conventional RCT. Several case reports have indicated the application of SGE on DI with pulpal or periapical diseases. The earliest report in 2013 used CBCT to produce plastic models of the tooth for training skills firstly, then prepared an external drilling-guide device for the access to the invagination cavity. This approach maintained the pulp vitality of the main root canal in the type IIIb DI whilst enabling the healing of the periapical tissues.^15^ Subsequent cases also manufactured static guides for the type II DI and allows endodontic treatment with precise and conservative pulp chamber access.^16,46,47^

Dens evaginatus (DE) is another type of anatomical abnormality with a tubercle, or supplemental solid elevation on some portion of the crown surface. DE is predominantly observed in Asian populations and often presents on the occlusal surface of mandibular premolars and lingual surface of anterior teeth.^48,49^ The pulp can extend to 70% of the tubercle, which is susceptible to pulpal horn exposure due to an occlusal erosion or brushing friction.^50^ Usually, DGT is unnecessary in majority of DE cases because the pulp is easy to be accessed. However, for the purpose of obtaining a minimally invasive buccal access, there is a case report using SGE on a central incisor with a tubercle on the medial gingival third and the medial buccal tooth surface.^51^

Dentin dysplasia (DD) is also a rare anatomical abnormality which causes accelerated dentin apposition. It is often characterized by normal enamel, atypical dentin formation, and narrowed pulp spaces. There are two subtypes of DD: DD-1, always appears normal shape and crown color, but is accompanied by sharp or absent root. DD-2, analogous characteristics of amber translucent crowns accompanied by significant attrition, with thin roots with normal length and obliterated pulp.^52,53^ The treatment plan of DD is decided by the dental history, age, pulp status, and root length. Once the teeth with DD and calcified canal is determined to undertake an RCT, DGT could be considered as an assist. For example, a case used SGE to locate obliterated root canals in six teeth of a patient with DD-1, and clear signs of apical healing were present at 1-year follow-up.^54^

#### Teeth need root canal retreatment

RCT usually fails when the treatment is carried out inadequately.^55^ In cases where a tooth necessitates root canal retreatment, the endodontist must reopen the tooth to eliminate the previous canal filling materials, which encompass not only the crown but also post and core materials, thereby enabling access to the root canals.^56^

Due to the aesthetics, high bonding capability, and similar elasticity modulus to dentin, fiber posts with a composite core are increasingly adopted to restore tooth structure.^57^ However, once the tooth needs a retreatment, the cement between post and dentin is hard to disrupt, and the fiber is difficult to be distinguished in the deep root canal even with the magnification of applying DOM. Routine method using ultrasonic tips and long-shaft burs for post removal is prone to cause a high prevalence of root perforation, axis deviation and consequent a poorer survival prognosis for the tooth.^58^ Employing SGE may help to remove the posts quickly and safely while minimizing the loss of the remaining tooth structure in anterior and posterior teeth.^59–63^

Recently, regenerative endodontic procedures (REPs) have been generally accepted as a treatment option for necrotic immature teeth. The steps of REPs contain intracanal medicaments, induction of bleeding, and placement of Mineral trioxide aggregate (MTA). If REPs fail or the teeth are traumatized, MTA barrier may need to be removed and RCT could be performed.^64^ In some teeth, mineralized tissues deposited in the canals and induced PCO after REPs in long-term inspection.^65^ Analogous to the post removal, MTA removal in the teeth previously treated by REPs may also result in excessive dentinal loss or iatrogenic deviation. An ex vivo study employed SGE for MTA removal and suggest it as a useful way,^66^ but no clinical report or study has been announced.

Moreover, in some cases, the treatment of PCO leads to iatrogenic deviation or perforation in root canals.^67^ Well-designed 3D guide seemed to be feasible for returning to the original canal and reaching patency during retreatment in some cases,^68–71^ but it should be clearly evaluated whether the canal relocation is worthy since DGT may furtherly weaken the tooth structure.

### EMS

EMS is a predictable alternative technique to nonsurgical treatment of persistent and recurrent periapical disease. The main purpose of EMS is to prevent bacterial leakage from the root canal system into the periapical tissues by placing root-end filling materials following root-end resection.^72^ Nonetheless, even with the aid of modern techniques including DOM, to precisely locating the root-end for resection and controlling the length of resection (3 mm) are challenging steps during EMS.^73,74^

Recently, SGE or DGE has been reported to be beneficial in navigating the exact location and resection of root-end in a minimally invasive way.^22,23,27,75^ As compared to the cases of anterior teeth with thin or defective buccal plate, GE seems to be more necessary than freehand (FH) in those cases surrounded by thick buccal plate and dangerous anatomical structures.

Trephine bur with an fixed external diameter (4 mm or 5 mm) was highly recommended due to its capacity to execute osteotomy and root-end resection in a single step, with predictable dimensions, angulation, diameter, and depth.^22^ TEMS using a hollow trephine bur could deal with the premolar or molar cases without damaging maxillary sinus, mental nerve, and greater palatine artery in a safe extent.^22,24,76^ A CBCT survey from 250 patients suggested that maxillary palatal root TEMS could be accomplished with a 2 mm safety margin in 47% of first molars and 52% of second molars because of greater palatine artery proximity and unfavorable resection angle or level.^77^

Moreover, one report used piezoelectric saw in the guided EMS,^78^ but the slowing cutting ability and large bone window that risk damaging neighboring teeth could be a matter of concern.^76^

### Tooth autotransplantation

Autotransplantation is a viable treatment option for a missing tooth when there is a donor tooth available in the same individual. It refers to the reposition of autogenous tooth in another tooth extraction site or surgically formed recipient site. Successful autotransplantation can offer a normally functioning periodontium, proprioception and preservation of alveolar bone volume.^79^ The intact and viable periodontal ligament cells, extra-oral time of the donor tooth, and the contact between the recipient site and root surface of the donor tooth are all critical elements, which affect prognosis of autotransplantation.^80,81^

European Society of Endodontology (ESE) has published a position statement on the background, procedure and outcome of surgical extrusion, intentional replantation and tooth autotransplantation in 2021, in which computer-aided rapid prototyping (CARP) models (tooth replicas) and 3D-printed guiding templates are suggested since they can provide the actual dimensions of the donor tooth and ideal 3D repositioning with reduced the extra-oral time and less of fitting attempts.^82^ The CARP model is used for practice before the surgical procedures or repeated fitting in the prepared bony socket in place of the real donor tooth.^83,84^ The surgical templates are printed for guided osteotomy preparation and donor tooth placement.^83,85^ To obtain most ideal 3D position and the required dimensions, multiple surgical templates or multi-drilling axis guides could be designed.^85–88^

### Intraosseous anesthesia

Intraosseous anesthesia is a supplemental technique of typical inferior alveolar nerve blocks, which allows the anesthetic solution to be injected directly into the cancellous bone.^89^ However, the technique-sensitive method is difficult to master as complications associated with the drill tip can arise, including inadequate perforation of the cortical plate, separation in the bone, and trauma to the adjacent periodontium or root.^90,91^ A preclinical study reported using dynamic navigation to deliver intraosseous anesthesia in 3D-printed jaw models. As compared to FH, DGE is safer in intraosseous drilling to prevent injury of the roots of the adjacent teeth in close proximity,^92^ yet it needs to be further explored in the clinical practice.

## General clinical workflow

### Pre-treatment considerations

The clinician should provide clear and elaborate information on the benefits and disadvantages of the treatment to patients. This enables patients to make an informed decision with regard to the treatment options proposed. Beforehand taken periapical radiograph is suggested, which represents the morphology and content of pulp canal with/without periapical lesions, thus provide the approximate anatomy and diagnosis to execute the treatment.

### General workflow of SGE

#### Extra-oral preparation

To make a precise template, case selection should be prudently. Clinically, SGE has been reported in dealing with RCT, EMS and tooth autotransplantation cases. Scattering in CBCT images could be produced by metallic restoration, which may compromise the accuracy of designed template. In some cases, clinicians could consider adding fiducial markers to overcome the limitation. For instance, an impression tray or scan appliance with small radiopaque gutta-percha could be worn during CBCT, then they can be removed and scanned by CBCT again. After paring the intra-oral and extra-oral CBCT files, the merged markers can accurately define the software’s alignment of files to provide more precise 3D construction.^30^

Commonly, a CBCT with a field of view (FOV) should be performed to clarify the detailed view of root, pulp canal, bone and adjacent neurovascular. Small FOV ( < 80 mm) CBCT is usually adequate for the diagnosis and management of endodontic diseases.^93,94^ Gauze or cotton rolls need to be placed between the teeth to prevent artifacts produced by maxillary and mandibular teeth touching.

OS is recommended for SGE in nearly all cases. If the static guide is designed by CBCT only, it may not allow for guide accommodation of soft tissues or tooth surface.^30^ OS can be performed in an intra-oral way directly,^13,23^ or an extra-oral way from impression and poured cast indirectly.^18,22^ The scan scope can be based on how much of the dentition will be covered in the guide to maintain the intraoperative stability.

OS is selective before surgery of tooth autotransplantation. It’s pointed out that guide and CARP models produced by CBCT merely showed acceptable accuracy,^95^ which was also feasible in clinical scenario.^96^

#### Template fabrication

CBCT Digital Imaging and Communications in Medicine (DICOM) files and stereolithography (STL) OS files should be both uploaded into the software which was designed for guided implantology purposes. To date, the study using customized GE software was very limited. After alignment of CBCT and OS files, a copy of the selected bur should be virtually superimposed. Then the designer may plan and check the ideal position of the drill. A virtual template is commonly provided with a guiding sleeve, which would be exported as an STL file. Ultimately, the template could be materialized through 3D printing or milling processes. Before autotransplantation, template for positioning of the donor teeth and CARP model of the donor teeth could be both fabricated.

#### Intra-oral procedure

Before RCT or root canal retreatment procedure in SGE, the fabricated template fitting should be checked first. Then the endodontic treatment could be initiated under local rubber dam isolation with or without anethesia.^13,33^ However, if the correct position of the guide is compromised, the rubber dam could also be applied after locating the root canal.^18,36^

A mark can be placed through the template sleeve and enamel could be removed by a diamond bur with high-speed handpiece until dentin is exposed. This step may be neglected in the root canal retreatment case. Then the cavity on the tooth will be precisely drilled by the selected bur using a pumping or pecking movement. The diameter, length, and rotation speed of drill should be determined by the occlusal distance, tooth type, and hardness in the canal. The reported diameter of burs varies from 0.8 mm to 1.5 mm, while the rotation speed is set differently from 350 rpm to 10,000 rpm.^13,17,18,20,33^ In a case of fiber post removal, the speed was even set at 40,000 rpm.^60^

The cavity should be copiously rinsed every 2 mm of the progression to avoid tooth overheating. When the apical target is reached, the tooth should be carefully examined under DOM. Upon location of root canal, standardized RCT is performed with instrumentation, irrigation, medication, obturation, and restoration.

Before applying SGE to EMS, the operator should evaluate whether drill bur insertion within its seated guide is obstructed by the patient’s cheek or contralateral dentition. After the template is printed, it should also be intra-oral fitted before surgery to verify precision. EMS will begin under local anesthesia and a full-thickness flap reflection.^21,23^ In some cases, EMS was performed by flapless technique or smaller flap design to avoid damage to important anatomic structures.^22,24,76^ The template should be positioned again, and guided osteotomy is performed. Then the exposed root surface would be checked after the template removal. The additional osteotomy, periapical curettage, root surface staining with methylene blue, root-end preparation with ultrasonic tips, retrograde filling, and suture would be performed routinely under DOM. TEMS technique using trephine bur may simplify this procedure because it can perform osteotomy and root resection simultaneously.^22,24^

During the process of guided autotransplantation, the fabricated guide is used for the preparation of recipient site, while the CARP model is used for pre-fitting of the donor tooth. The details could be followed according to the official guidelines of ESE.^82^

**Fig. 1:**
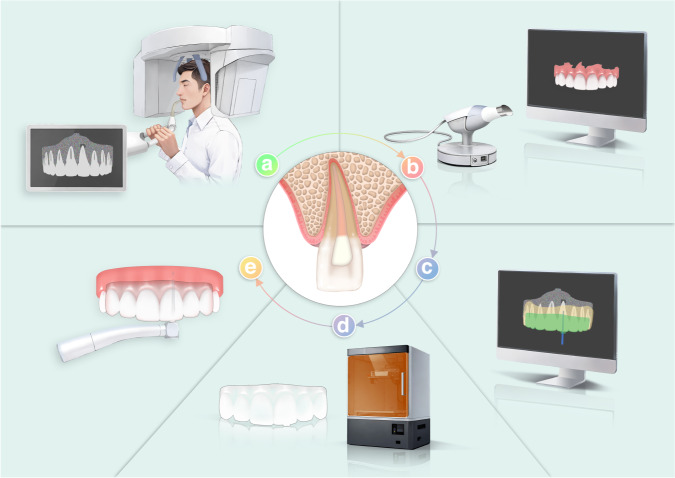
Schematic diagram of SGE **a**. CBCT scanning **b**. Oral scanning **c**. Virtual guide design **d**. Template printing **e**. Intra-oral guided drilling

The general workflow of SGE is exhibited in Fig. 1.

### General workflow of DGE

#### Extra-oral preparation

Since no templates are required in DGE, OS is unnecessary for the DGE procedure. Significantly, the operator must have adequate ex vivo practice for acquiring a hand-eye coordination of DGE.^27,28^ DGE has been reported in dealing with RCT and EMS, but whether it could be used for the autotransplantation still needs to be testified.

Before taking CBCT, a registration device with radiopaque fiducial markers should be placed on the teeth on contralateral side of the dentition first. Then a CBCT should be scanned, with the DICOM images uploaded to a DNS. The virtual drilling path could be designed by the embed software in the system.

According to the manufacturer’s instruction, the calibration between the handpiece and the registration device must be performed. Then the registration device should be reinserted to the teeth, so the paring between CBCT fiducial and the intra-oral position under the camera can produce the transition matrix to complete the eye-hand calibration. The tracking software will allow the clinician to get live feedback to visualize the location, angle, and depth of drilling.

#### Intra-oral procedure

In the process of DGE, the clinicians just need to drill and adjust the location, angle, and depth in real time to access the target with visualizing the virtual bur on the screen of the system.^28,35,76,97^ Flap design should also be based on the tooth type, tooth location, and gingiva type in EMS, while round diamond bur and trephine bur could both be employed in the surgery.^27,75^

**Fig. 2:**
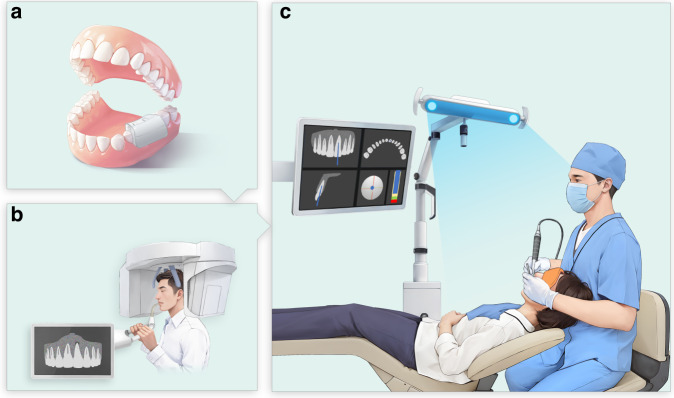
Schematic diagram of DGE **a**. Registration device placement **b**. CBCT scanning **c.** Dynamic navigation after calibration

The general workflow of DGE is exhibited in Fig. 2.

## Evaluation

Basic researches all indicate that DGT as a high-efficiency, minimally invasive, and accurate way as compared to FH. Disregard for those merits mentioned above, the advantage of DGT must be carefully balanced against a greater radiation burden, higher costs, and more difficult debridement and visualization of the pulp chamber and root canals.^98^ The application of DGT is also limited in posterior teeth due to the tracing and operative difficulty. Besides, DGT technique still needs to be improved for curved canals to avoid the high risk of perforation.

To date, very limited evidence can verify whether SGE or DGE is more applicable for clinical use. It’s claimed that the success of the SGE may not be influenced by the experience of the operator,^99^ but the outcome of DGE seemed to be dependent of operator’s experience. For instance, more experienced clinician can achieve less substance loss.^100^

### RCT

#### Duration ex vivo

An ex vivo experiment stated that the mean duration of SGE including OS, virtual planning, design of template, removal of enamel, and preparation of access cavity was 10 min on mandibular anterior tooth.^34^ When applied to incisors with simulated calcified canals, the treatment time of SGE lasted 11.3 min, and it needed 21.8 min for the conventional technique.^99^ Although the duration of SGE is decreased, the studies didn’t calculate the extra time of 3D printing.

The average drilling time of DGE was 57.8 s with significant dependence on the canal orifice depth, tooth type, and jaw. The maximal duration was 136.7 s on maxillary anterior.^101^ A research on 3D printed incisor with calcified root canals also showed the similar access preparation time using DGE was 2.2 min, while it needed 7.06 min in FH group.^102^ However, another study claimed no significant difference was found between mean treatment time of DGE group with FH group (195 s vs 193 s), which may be affected by the operator’s experience.^100^ Particularly, DGE has a learning curve and needs extensive training prior to its clinical application. Besides, the DNS may struggle to recognize the attached drill tag in molars, yet this problem can be resolved by drill tag redesigning.^26^

#### Accuracy ex vivo

Basic studies support that the accuracy of access cavity preparation by DGT was acceptable. However, the average linear deviations of the anterior teeth and premolars were significantly lower than molars when utilizing SGE, which was possibly caused by the deviated entry point of the bur due to the interference of the opposite teeth.^103^ DGE could also increase the benefits of ultra-conservative access cavities by preserving critical structures of the crown and reducing negative influences to shaping procedures.^104^ In addition, a study on single rooted premolars indicated that SGE was more beneficial than FH for preserving the periodontium because of a lower root surface temperature rise.^105^ The accuracy, tooth structure loss, and success rate related to different DGT methods on RCT or retreatment were listed in Table 1.

**Table 1:** Assessment of accuracy, tooth structure loss, and success rate on RCT or retreatment by different DGT methods ex vivo

Order	Authors	Samples	Method	Mean linear deviation/mm	Mean angular deviation/°	Substance Loss/mm^3^	Success rate/%
1	Buchgreitz J, et al.^126^	48 teeth mounted in acrylic blocks	SGE	<0.7 mm	N/A	N/A	N/A
2	Zehnder MS, et al.^14^	60 single rooted teeth in maxillary models	SGE	mesial/distal (base) 0.21 buccal/palatal(base) 0.2 apical/coronal(base) 0.16 mesial/distal (tip) 0.29 buccal/palatal (tip) 0.47 apical/coronal (tip) 0.17	1.81	N/A	100
3	Connert T, et al.^34^	60 anterior teeth in mandibular models	SGE	mesial/distal (base) 0.12 buccal/oral (base) 0.13 apical/coronal(base) 0.12 mesial/distal (tip) 0.14 buccal/oral (tip) 0.34 apical/coronal (tip) 0.12	1.59	N/A	100
4	Zhang C, et al.^105^	40 single-rooted premolars in epoxy model	SGE	mesial/distal (base) 0.28 buccal/lingual (base) 0.25 mesial/distal (tip) 0.30 buccal/lingual (tip) 0.28	3.62	N/A	100
5	Su Y, et al.^103^	36 anterior teeth, 24 premolars, and 24 molars in maxillary and mandible models	SGE	anterior (base) 0.09 premolar (base) 0.07 molar (base) 0.22 anterior (tip) 0.28 premolar (tip) 0.40 molar (tip) 0.64	anterior 1.73 premolar 2.23 molar 4.00	N/A	71.8
6	Connert T, et al.^99^	48 printed incisors with calcified canals	SGE	N/A	N/A	9.8	91.7
			FH			49.9*	41.7*
7	Loureiro MAZ, et al.^127^	20 mandibular incisors and upper molars	SGE	N/A	N/A	incisor 26.5 molar 45.7	N/A
			FH			incisor 31.7 molar 62.5*	
8	Kostunov J, et al.^98^	typodont teeth with 30 canals in acrylic resin model	SGE	N/A	N/A	incisor 10.3 premolar 29.3 molar 51.8	N/A
			FH			incisor 16.1* premolar 44.2* molar 99.3*	
9	Jain SD, et al.^101^	84 printed teeth with calcified canals in maxillary and mandibular models	DGE (Navident)	anterior (base) 1.0 premolar (base) 1.2 molar (base) 1.0 anterior (tip) 1.3 premolar (tip) 1.1 molar (tip) 1.4	anterior 1.53 premolar 1.38 molar 1.89	N/A	100
10	Torres A, et al.^128^	132 printed teeth with calcified canals in maxillary or mandibular models	DGE (Navident)	anterior 1.77 premolar 1.54 molar 1.37	anterior 2.68 premolar 2.73 molar 3.01	N/A	93
11	Gambarini G, et al.^104^	132 artificial teeth in silicon bases	DGE (Navident)	0.34	4.8	N/A	N/A
			FH	0.88^	21.2^		
12	Dianat O, et al.^129^	60 single-rooted teeth with calcified canals in cadaver jaws	DGE (X-guide)	mesial/distal 0.12 buccal/lingual 0.19	2.39	N/A	96.6
			FH	mesial/distal 0.31^ buccal/lingual 0.81^	7.25^		83.3
13	Jain SD, et al.^102^	40 3D printed teeth with calcified canals in maxillary or mandibular models	DGE (Navident)	N/A	N/A	Maxilla 35.5 Mandible 19.0	90
			FH			Maxilla 62.2^ Mandible 19.1	85
14	Connert T, et al.^100^	72 typodont teeth on models	DGE (DENACAM)	N/A	N/A	10.5	97.2
			FH			29.7^	97.2
15	Zubizarreta-Macho Á, et al.^130^	30 single rooted anterior teeth in epoxy resin models	SGE	base 7.44 tip 7.13	10.04	N/A	N/A
			DGE (Navident)	base 3.14 tip 2.48	5.58		
			FH	base 4.03*^ tip 2.43*^	14.95*^		
16	Ali A, et al.^66^	30 mandibular premolars with MTA placement at 3 mm below CEJ	SGE	N/A	N/A	N/A	100%
			FH				86.7%*
17	Perez C, et al.^63^	40 teeth with RCT, fiber posts, and composite build-ups	SGE	mesial/distal (coronal) 0.28 buccal/oral (coronal) 0.23 global (coronal) 0.39 mesial/distal (apical) 0.26 buccal/oral (apical) 0.24 global (apical) 0.40	N/A	N/A	87.5%
			FH				
18	Janabi A, et al.^62^	26 maxillary teeth with RCT, fiber posts, and core build-ups	DGE (X-guide)	global (coronal) 0.91 global (apical) 1.17	1.75	54.63	N/A
			FH	global (coronal) 1.13* global (apical) 1.68*	4.49	38.18	

SGE static guided endodontics, DGE dynamic guided endodontics, FH freehand, MTA mineral trioxide aggregate, CEJ cementoenamel junction, *P < 0.05 as compared to SGE, ^*P* < 0.05 as compared to DGE

#### Clinical performance

Besides the case reports, only one case-series study assessed the clinical performance of SGE when performed on calcified single-rooted teeth in 50 patients. The results indicated that the cases were all successful clinically. The drill path in mandibular teeth acquired higher optimal precision scores than that in maxillary teeth. A previous attempt at access and canal negotiation, which may reduce the resistance to the bur to the obliterated part, also showed higher optimal precision scores than that with no attempt.^106^

### EMS

#### Duration ex vivo

The introduction of DGT could save the operation time in the surgery procedure, which may reduce the iatrogenic risk of swollen and delayed healing.

The time from bone fenestration to root-end resection was approximately 155.71 s and 189.75 s in experienced and inexperienced operators, respectively. Although it was slightly faster in SGE, there was no significant difference when compared to FH.^107^ The duration may be significantly reduced when utilizing special surgery plans for SGE. For instance, when SGE was supplemented by a fully guided drill protocol, mean time for osteotomy and root end resection in SGE was 140 s, which was significantly less than FH (604 s).^108^ TEMS utilizing trephine bur also significantly reduced clinical measurement and surgical time from an average of 943 s to 293 s.^25^

Applying DGE for osteotomy and root end resection required 800 s, while employing FH needed 1423 s.^109^

#### Accuracy ex vivo

Basic studies support the accurate root resection, the minimal tissue removal, and the excellent success rate by DGT. Data from various research groups are listed in Table 2. Importantly, mishaps including sinus perforation or incomplete root-end resection may either occur during SGE or DGE, which may be caused by improper placement of the template or indirect view of the surgical field, respectively.^108,110^

**Table 2:** Assessment of accuracy, tooth structure loss, and success rate on EMS by different methods ex vivo

Order	Authors	Samples	Method	Mean linear deviation/mm	Angle/°	Tissue removal/mm^3^	Success rate/%
1	Pinsky HM, et al.^131^	10 dry mandibles with full set of teeth	SGE	apex 0.79	N/A	N/A	<3 mm 100
			FH	apex 2.27*			(dt) <3 mm 76 (dt)
2	Ackerman S, et al.^111^	48 roots in cadaver model	SGE	apex 1.473	N/A	N/A	<4 mm 100 (dt)
			FH	apex 2.638*			<4 mm 45.8 (dt)
3	Peng L, et al.^107^	56 maxillary anterior teeth in gypsum model fixed on the head-simulator	SGE	apex (ex) 0.31 apex (in) 0.31	deviation(ex) 5.04 deviation(in) 6.79	N/A	N/A
			FH	apex (ex) 0.99 * apex (in) 1.18*	deviation(ex) 16.74* deviation(in) 15.06*		
4	Hawkins TK, et al.^25^	72 teeth on 3D-printed Maxillary and mandibular models	TEMS	N/A	resection 6	bone 58.2 root 27.2	N/A
			FH		resection 10.6*	bone 54.9 root 38.3*	
5	Westbrook K, et al.^108^	46 roots on cadaver heads	SGE	platform 1.31 apex 1.49	deviation 1.82 resection 2.9	N/A	no perforation 95.7
			FH	platform 2.59* apex 3.15*	deviation 10.3* resection 8.3*		no perforation 95.7
6	Aldahmash SA, et al.^109^	48 roots on cadaver heads	DGE (X-guide)	platform 0.6 apex 1.07	deviation 1.1 resection 9.05	bone 82.4	N/A
			FH	platform 1.29^ apex 2.57^	deviation 16.03^ resection 21.12^	bone 125.2^	
7	Dianat O, et al.^110^	40 roots on cadaver heads	DGE (X-guide)	platform 0.7 apex 0.65	deviation 2.54	N/A	no mishaps 90%
			FH	platform 2.25^ apex 1.71^	deviation 12.38^		no mishaps 80%
8	Tang W, et al.^114^	64 teeth on 3D printed maxillary models	SGE	length (ex) 0.20 length (in) 0.26 depth (ex) 0.65 depth(in) 0.71	deviation (ex) 3.23 deviation (in) 4.08	ex 3.39 in 3.70	no mishaps 100%
			DGE (DCARER)	length (ex) 0.21 length (in) 0.28 depth (ex) 0.45 depth(in) 0.53	deviation (ex) 6.34 deviation (in) 7.18	ex 3.36 in 3.75	no mishaps 100%
			FH	length (ex) 0.68*^ length(in) 1.21*^ depth (ex) 1.36*^ depth(in) 1.91*^	deviation (ex) 16.2*^ deviation (in) 20.45*^	ex 6.70*^ in 10.78*^	no mishaps (ex) 91.7 no mishaps (in) 58.3*^
9	Martinho FC, et al.^132^	50 roots on cadaver heads	DGE (X-guide)	platform 1 apex 1.14	deviation (1.94) resection (5.66)	bone 82.27	96%*
			SGE	platform 1.15 apex 1.21	deviation (1.70) resection (4.70)	bone 76.22	80%^

SGE static guided endodontics, TEMS targeted endodontic microsurgery, DGE dynamic guided endodontics, FH freehand, ex experienced, in inexperienced, an anterior, po posterior, dt distance from the target, *P < 0.05 as compared to SGE, ^*P* < 0.05 as compared to DGE

#### Clinical performance

DGT shows an especial feasibility in those problematic cases of EMS. Clinicians can perform EMS on posterior teeth with the assistance of DGT, and the accuracy or efficacy of the root-end resection may not be impacted by thick buccal cortical plate.^110^ Moreover, the fabricated template in SGE can serve as a passive reflector for reflected flap, which may minimize trauma to soft tissue.^111^

A retrospective study showed that the success rate of TEMS of 24 cases was 91.7% at 1 year or beyond by radiograph and clinical examination, in which 70.8% of cases were presented with anatomic complexities.^77^ Another study performed on 11 teeth in 9 patients also indicated a high accuracy and acceptable success rate (90%) of DGE at 1 year or beyond. The platform and apex deviation were significantly less in the posterior teeth as compared to the anterior teeth, but the second or third molar were excluded.^29^

It is worth mentioning that a randomized controlled trail is in progress to compare the clinical outcomes of the DGE and FH.^112^

### Tooth autotransplantation

The combined utilization of guide and CARP model is a useful option of autotransplantation that involves minimal bone preparation in a short surgical time. The mean angular deflection of donor teeth with the planned position was 5.6°, and the mean deviation at the shoulder/apical position was 3.15 mm/2.61 mm ex vivo.^113^

Clinical report using multidrilling axis guide and CARP showed that all the 10 transplanted teeth fulfilled the criteria for success over a mean follow-up time of 13.1 months. No signs of progressive root resorption or pain were observed. When compared to conventional FH technique with a success rate of 78%, using guides achieve a clinical success rate of 86% within a mean follow-up period at 4.5 years. Although there was no significant difference between two groups, the method could reduce the number of repeated attempts of positioning the donor teeth, as well as controlling the extra-oral time of donor tooth and the total surgery time. It is notable that failure including ankylosis with replacement resorption or periapical infection caused by subsequent caries may still happen in template guided group, but inflammatory root resorption and external cervical root resorption only occurred in FH group.^96^

## Influence factors

In summary, influence factors related to accuracy of DGT may be divided by three aspects, which include the operators’ error depended on their proficiency, the radiographic error depended on CBCT image quality, and the system errors from equipment and software.

### Clinician’s experience

It’s widely accepted that experienced clinician exhibits a more precise and efficient clinical procedure. Current evidence could not verify that SGE may improve the chairside efficiency of operators. For example, one laboratory study demonstrated that there was no significant difference between experienced and inexperienced clinicians regarding to the operation time in osteotomy and root resection during SGE, yet no significant difference was found during FH, either.^107^

In another study, SGE significantly improved the efficiency of both operators, while DGE seems to increase the accuracy of the inexperienced operator.^114^ DGE also helps inexperienced operator to obtained less substance loss in access cavity preparation as compared to FH.^100^ Moreover, the benefit of DGE could be enhanced on more experienced operators. In a cadaver study, DGE could improve the accuracy of both experienced and inexperienced endodontists as compared to FH, but it didn’t allow inexperienced endodontists to perform osteotomy and root end resection as precise as experienced endodontists.^115^

### Radiographic quality

The image quality of CBCT may be decided by FOV, voxel size, exposure time, and other technical elements. FOV and voxel size varies in clinical cases reports of GE. Few studies have been carried to explore the effect of CBCT on GE. Recently, an in vitro study stated that CBCT with different FOV (80 mm, 60 mm, and 40 mm) and voxel size (0.3 mm, 0.16 mm, and 0.08 mm) did not play a critical role in the accuracy of DGE in EMS. Considering the image quality and radiation dose, the operator should select a limited FOV to cover the registration device, involved teeth, and periapical lesion. In addition, the voxel size should be determined based on the required resolution and units.^116^

### Systematic difference

3D printer is a key equipment during SGE. Print quality may be influenced by the size of the model, postprocessing, the capabilities of printers, layer height, and the build speed. The printers are categorized to 3 types utilizing fused deposition modeling (FDM), digital light processing (DLP), and stereolithography (SLA) techniques, respectively. FDM was not recommended in GE because of the unsatisfied print quality. A study found that either DLP or SLA technique could produce templates that allowed high accuracy in canal localization of the artificial tooth. However, statistically significant differences existed among the printers regarding to the axial deviation of SGE.^117^

Registration is a critical step in DNS for spatial connection between the virtual plan and software. Marker point-based methods including U-tube embedded with radiopaque fiducial markers are widely used for registration, but it may be difficult to position on tooth with short crown or shallow vestibule. Thus, tooth cusp registration could serve as an alternative way in implant surgery and exhibits similar levels of accuracy as compared to U-tube.^118^ However, when it was applied to EMS, it is still less precise and efficiency than U-tube registration, yet it didn’t need an additional registration device.^119^

## Conclusion and expectation

AS compared to FH, DGT is a practicable and time-saving method for guiding obliterated/deformed root canal location, EMS within dangerous area or thick cortical bone plate, and autotransplantation with ideal position. Despite these profits, the current procedure for DGT needs to be critically questioned. On the one hand, preoperative CBCT is mandatory for current DGT procedure, which brings the ionizing radiation burden to patients. The costs of template fabrication or DNS also aggravate the economic burden for both patients and clinicians. On the other hand, the procedures of DGT including restricted drilling through obstructed view of template and hand-eye coordination of DGE are challenging for most clinicians even with specialized training. In addition, the clinical applications of DGT are focused on case reports which still need to be further verified with its advantage by randomized controlled clinical trials in future.

Recently, magnetic resonance imaging (MRI) based GE has been developed for access cavity preparation and show comparable accuracy as CBCT-based GE.^120^ Augmented reality (AR) technique has also been introduced to enhance the clinician’s view by displaying and matching images and digital guides to the patient’s anatomy.^121–123^ In dental implant surgery, robot-assisted systems are developed and offer haptic guidance for implant treatment planning, osteotomy preparation, and implant placement, which may provide a novel strategy for improving the stability of human hands.^124^ Additionally, artificially intelligence (AI) is emerging in medical domain including endodontics. AI may help with diagnosis and treatment that can be combined to DGT for increasing precision and convenience of endodontic treatment.^125^

In conclusion, DGT already causes an improvement in the success of endodontic treatment outcomes. However, the clinical practice still requires verification in terms of its reliability, applicability, and cost-effectiveness. More economical and practical systems should be designed especially for promoting DGT in endodontics. The ongoing evolution of technology offers promising avenues to further improve and refine DGT, shaping the landscape of modern endodontic practice.
